# Predicting of diabetic retinopathy development stages of fundus images using deep learning based on combined features

**DOI:** 10.1371/journal.pone.0289555

**Published:** 2023-10-20

**Authors:** Ahlam Shamsan, Ebrahim Mohammed Senan, Hamzeh Salameh Ahmad Shatnawi

**Affiliations:** 1 Computer Department, Applied College, Najran University, Najran, Saudi Arabia; 2 Department of Artificial Intelligence, Faculty of Computer Science and Information Technology, Alrazi University, Sana’a, Yemen; University of Mauritius, MAURITIUS

## Abstract

The number of diabetic retinopathy (DR) patients is increasing every year, and this causes a public health problem. Therefore, regular diagnosis of diabetes patients is necessary to avoid the progression of DR stages to advanced stages that lead to blindness. Manual diagnosis requires effort and expertise and is prone to errors and differing expert diagnoses. Therefore, artificial intelligence techniques help doctors make a proper diagnosis and resolve different opinions. This study developed three approaches, each with two systems, for early diagnosis of DR disease progression. All colour fundus images have been subjected to image enhancement and increasing contrast ROI through filters. All features extracted by the DenseNet-121 and AlexNet (Dense-121 and Alex) were fed to the Principal Component Analysis (PCA) method to select important features and reduce their dimensions. The first approach is to DR image analysis for early prediction of DR disease progression by Artificial Neural Network (ANN) with selected, low-dimensional features of Dense-121 and Alex models. The second approach is to DR image analysis for early prediction of DR disease progression is by integrating important and low-dimensional features of Dense-121 and Alex models before and after PCA. The third approach is to DR image analysis for early prediction of DR disease progression by ANN with the radiomic features. The radiomic features are a combination of the features of the CNN models (Dense-121 and Alex) separately with the handcrafted features extracted by Discrete Wavelet Transform (DWT), Local Binary Pattern (LBP), Fuzzy colour histogram (FCH), and Gray Level Co-occurrence Matrix (GLCM) methods. With the radiomic features of the Alex model and the handcrafted features, ANN reached a sensitivity of 97.92%, an AUC of 99.56%, an accuracy of 99.1%, a specificity of 99.4% and a precision of 99.06%.

## 1. Introduction

**Fig 1:**
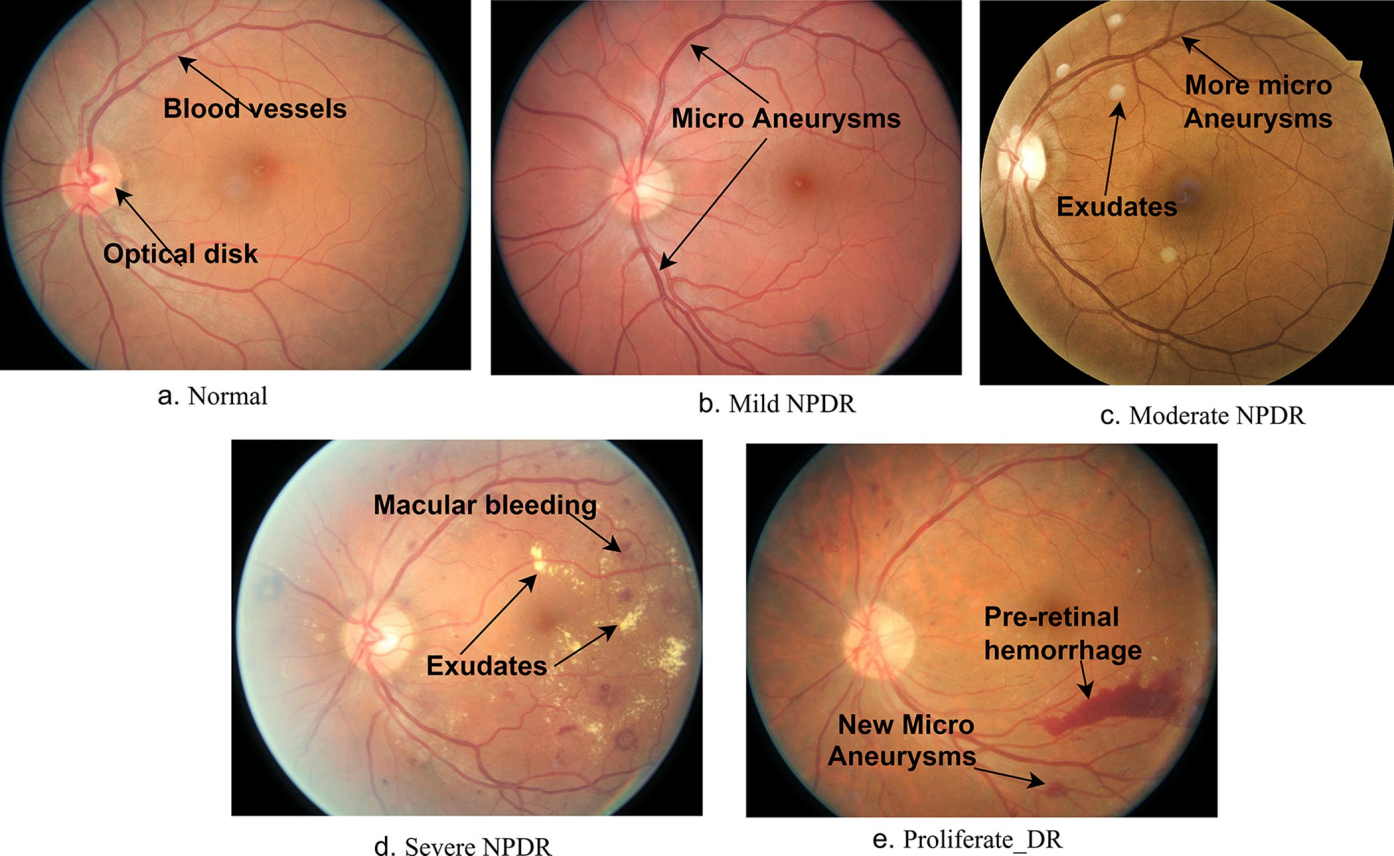
DR stages of development by the appearance of features.

Early detection of any disease is considered effective in providing appropriate patient treatments and curing them. A retina is a group of thin tissues responsible for vision. The retina receives the light and converts it into nerve signals that it sends to the brain. In diabetic patients, the amount of glucose in the blood rises in due to a lack of insulin, which causes damage to the retina of the eye, which is called DR [1]. High blood pressure in the eye or diabetes causes damage to the small blood vessels in the retina due to abnormal blood flow and causes blindness. According to the World Health Organization (WHO), as of now, diabetes is not listed among the leading causes of death. However, it is projected to become the seventh leading cause of death by 2040. Furthermore, the number of individuals affected by diabetes is expected to reach 642 million, with an estimated one-third of them developing diabetic retinopathy (DR). This highlights the urgency of the situation and the concerning trend for the future [2]. Diabetes affects the heart, kidneys, retina and nerves [1]. DR is one of the complications of diabetes and causes the micro-vascular to swell and explode and goes through several stages until it reaches the advanced stage, which leads to blindness [3]. The percentage of people who are blind due to DR is 2.6% [4]. The more prolonged diabetes, the higher risk of developing DR. Therefore, it is necessary to examine the retina regularly for diabetic patients to detect DR in its early stages to avoid its development to advanced stages in which the patient becomes blind [5]. DR is detected by the appearance of some variety of lesions in the retina. These diverse lesions include Microvascular Aneurysms (MA), hard and soft exudate and hemorrhagic [6]. The first early sign of DR is MA, which appears as small, circular red dots about 120 μm in size with sharp margins. Hard exudates in the retina, due to plasma leakage, leads to the appearance of bright yellow spots with sharp margins in the outer layers. Soft secretions due to swelling of the nerve fibres lead to the appearance of white, circular and oval-shaped spots. Hemorrhage in the retina shows spots larger than 125 μm with irregular margins. There are two types of retinal hemorrhage, superficial and deep. The DR passes through five stages based on the presence of the previously mentioned lesions, which are no DR, mild DR, moderate DR, and severe DR. These four stages are called non-proliferative DR (NPDR). In contrast, the last and most serious stage is DR Proliferative. Fig 1 shows a picture of each stage with the appearance of the lesions (vital signs) for each stage. The lesions that appear in red are MA and HM, while the other ones that appear in yellow and white are hard and soft secretions. Each year, 10% of patients with diabetes who do not have DR will be infected with the first stage of DR. The patients with Severe NPDR will be infected with the Proliferative Diabetic Retinopathy (PDR) fourth stage by 75% annually. Each stage of DR requires a different treatment than the other stage. People with diabetes without DR or Mild DR need regular checkups. While patients with Moderate DR (second stage) and Severe DR (third stage) require appropriate laser treatment or vitrectomy. The PDR forms abnormal blood vessels that rupture and bleed, leading to blindness. Therefore, the diagnosis of the first three stages, called NPDR is more effective in avoiding reaching the stage of PDR. With the increasing number of diabetic patients, the regular diagnosis of patients requires a large number of skilled ophthalmologists. It requires time and effort to detect DR and determine its stage. Manual diagnosis is also more prone to errors and differing opinions of doctors. Therefore, automated diagnosis by artificial intelligence techniques saves time and effort and gives more accurate results than manual diagnosis [10]. Because of the similarity of features in the stages of DR, this study reviews several hybrid techniques that rely on the mixed features of several methods for early diagnosis of the stages of development of DR.

The main contributions to this work are as follows:

- Image processing using the average filter and the CLAHE method to increase the contrast of microvascular, white and yellow spots and eliminates noise.
- The features of the Dense-121 and Alex models are passed to the PCA method to select the important features and reduce their dimensions.
- Integrating the features before and after reducing its high dimensions and feeding it to the ANN to diagnose DR.
- Integrating features of DWT, LBP, FCH, and GLCM methods to produce handcrafted features.
- Integrating the features of the Dense-121 and Alex models separately with the handcrafted features to produce the radiomic features and feed them to the ANN to diagnose.
- Developing effective systems to assist ophthalmologists and experts with their diagnoses and determination of the stage of DR.

The rest of the paper is organized as follows: Section 2 discusses a set of techniques and results from previous studies. Section 3 reviews methodologies for analyzing colour fundus images for early prediction of DR disease. Section 4 summarizes the performance of the systems and presents their results for diagnosing colour fundus images of the DR dataset. Section 5 discusses the performance of the methods and compares their impact on the diagnosis of colour fundus images of the DR dataset. Section 6 concludes the work.

## 2. Related work

Many experts in the field of artificial intelligence have devoted their time and effort to detect the early stages of DR disease development. The researchers applied various techniques with the aim of achieving satisfactory accuracy. Our study features hybrid techniques with hybrid features to detect DR disease and determine its stage development.

Saxena et al. [7] presented a deep learning network based on machine learning methods for the retinal image dataset. The model was trained on the EyePACS dataset and tested on the Messidor-2 and Messidor-1 datasets. The system achieved AUC and sensitivity of 92% and 81.02% on the Messidor-2 dataset and AUC and sensitivity of 95.8% and 88.84% on the Messidor-1 dataset. Abhishek et al. [8] proposed a lightweight CNN architecture to diagnose colour fundus images. The method yielded good results with a more minor data set than skewed classes. The Cohens Kappa model has the right of 88.36% for the validation data set. The system achieved a Kappa score of 0.8836 on the validation data and 0.9809 on the training data. Borys et al. [9] designed a CNN-based method for detecting stages of DR progression on retinal fundus images. Images are optimized and data augmented for pre-trained models to get good results. The EfficientNet-B4 achieved an accuracy of 90.3%, sensitivity of 80.12% and specificity of 97.6%. Muhammad et al. [10] Development of a framework for a pre-trained CNN model for retinal images diagnosis. Retinal images were optimized for exposing the abnormal exudate, ROI localization, and feature extraction specify the three pre-trained models. Silky et al. [11] trained CNN models and machine learning algorithms on DR images to diagnose them before they progressed to dangerous stages. The Convolutional Neural Network (CNN) and Random Forest (RF) method achieved an accuracy of 90.2% and 86.1% and a Recall of 77.3% and 95.8% for diagnosing Class Soft Exudate (SE). Shu et al. [12] propose a CNN with two-channel to diagnose retinal images based on green and gray colour according to the entropy scale. Before calculating the entropy, the images were optimized by blunt masking to improve detection accuracy. The CNN achieved an accuracy of 87.83%, sensitivity of 77.81% and specificity of 93.88%. Alexandr et al. [13] proposed a network that uses fewer resources by comparing the DenseNet and ResNet models with the improved EfficientNet architecture. The two models were applied to the APTOS data set for retinopathy diagnosis. EfficientNet-b4 achieved an accuracy of 65.6% before enhancement, while the accuracy after enhancement was 69%. While ResNet101 reached an accuracy of 60.7% before enhancement, the accuracy after enhancement reached 65%. Fouzia et al. [14] presented machine learning methods based on deep learning to diagnose retinopathy. The Inception model uses two transfer learning variables by setting parameters and extracting features. The Inception model with fine composition tuning achieved an accuracy of 96.6%. Gadekallu et al. [15] proposed a DNN model with the Firefly method to diagnose the retinopathy dataset. The data set was normalized and the most important features were extracted by PCA and then the dimensions were reduced by the Firefly method. The model achieved an accuracy of 90.07% and a sensitivity of 87%. Ludwig et al. [16] proposed a pre-trained deep-learning model to diagnose the retinopathy dataset. Images have been improved, and images augmented to eliminate the overfitting problem. The method achieved a specificity of 83% and a sensitivity of 89%. Ayushi et al. [17] proposed a image processing and machine learning methods for detecting DR for the DIARETDB dataset. The technique focused on improving the images, extracting features, and applying machine learning methods to classify retina images. Abnormal microvascular edges and secretions were detected, with KNN and Simple Tree algorithms achieving an accuracy of 85.8% and 88.6%. Nneji et al. [18] A framework uses two channels of fundus images to identify retinopathy symptoms. The features of the images are extracted using fine-tuned Inception V3 and VGG-16, respectively. The outputs of the two models are merged and classified using softmax. The framework achieves an accuracy of 98.5%, sensitivity of 98.9%, and specificity of 98.0% on the Messidor dataset. The framework achieves an accuracy of 98.0%, sensitivity of 98.7%, and specificity of 97.8% on the Kaggle dataset. Renukadevi et al. [19] presented a density-related GoogLeNet model for detecting DR from the APTOS data set. The method was implemented in several stages: preprocessing, modelling, data augmentation, feature extraction, and classification through the last layer in a model. The DenseNet-169 model achieved an accuracy of 86.8%. Laxmi et al. [20] proposed a pre-trained adaptive CNN based on a segmented learning approach. The approach mutually learns the features from the images and gets a good performance to recognize the colour fundus images of DR. CNN works at the segment level and then all segments are combined at the final classification. The method achieved a sensitivity of 96.37% and an AUC of 96.3%. Gadekallu et al. [21] designed a DNN model based on selecting optimal features by GWO algorithm to diagnose the DR images. The data set images were normalized, then dimensionally reduced, and finally the data set was trained and evaluated by DNN. The model achieved a sensitivity of 91% and an accuracy of 97.3%.

## 3. Materials and methods

**Fig 2:**
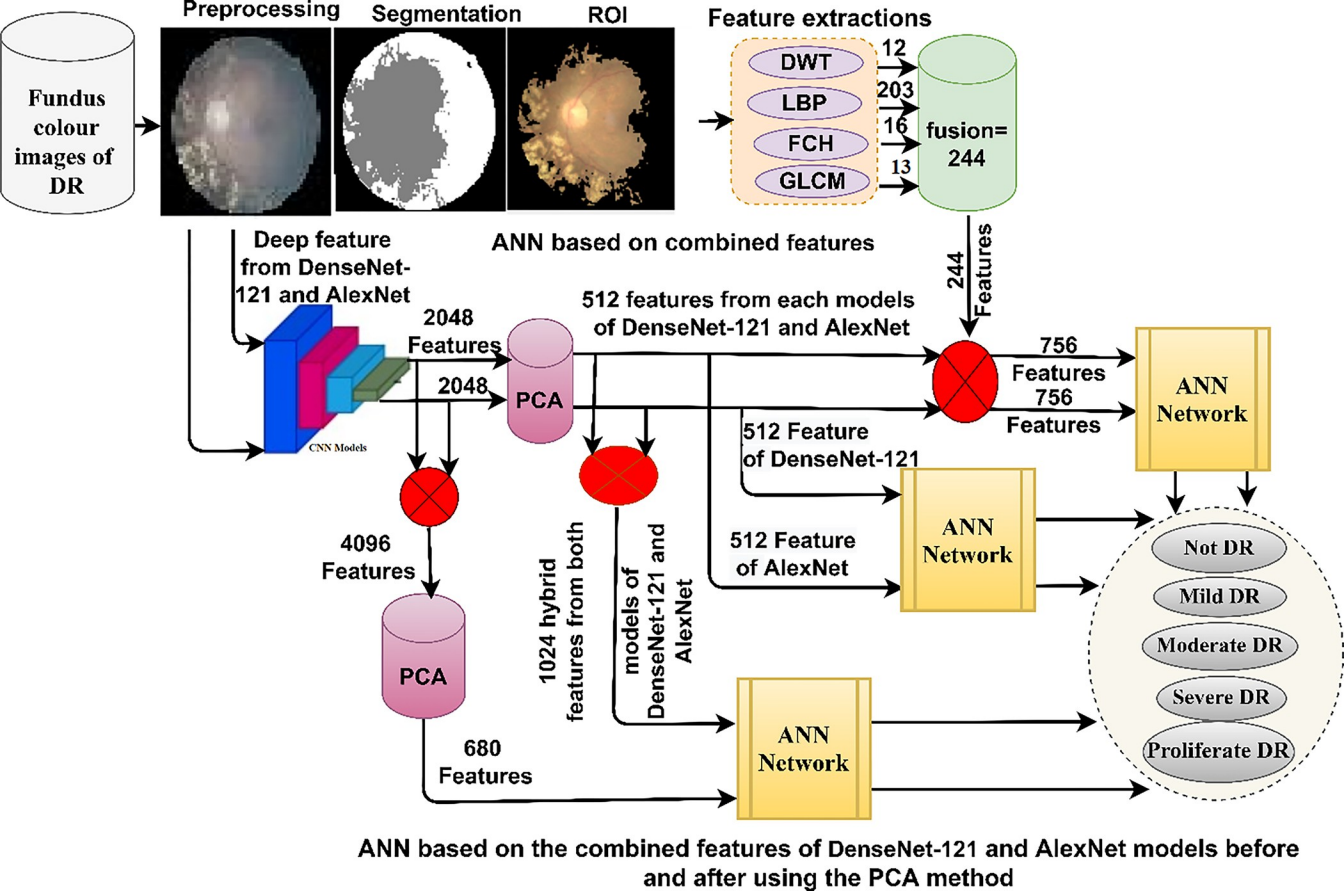
Structure of the fundus colour images analysis for early diagnosis of diabetic retinopathy in its early stages.

This section presents methodology and materials for diagnosing colour fundus images for early prediction of DR in its early stages before blindness as shown in Fig 2. All images have passed optimization to increase ROI contrast and remove noise. All features resulting from Dense-121 and Alex models were subjected to dimensionality reduction and selection of important features by PCA. First, the images of the DR dataset were diagnosed by ANN with significant features of Dense-121 and Alex. Secondly, DR dataset images were diagnosed by ANN with hybrid features of Dense-121 and Alex low-dimensional before and after PCA. Third, the DR dataset images were diagnosed by ANN with radiomic features of Dense-121 and Alex models with handcrafted features.

### 3.1. Description of the DR dataset

**Fig 3:**
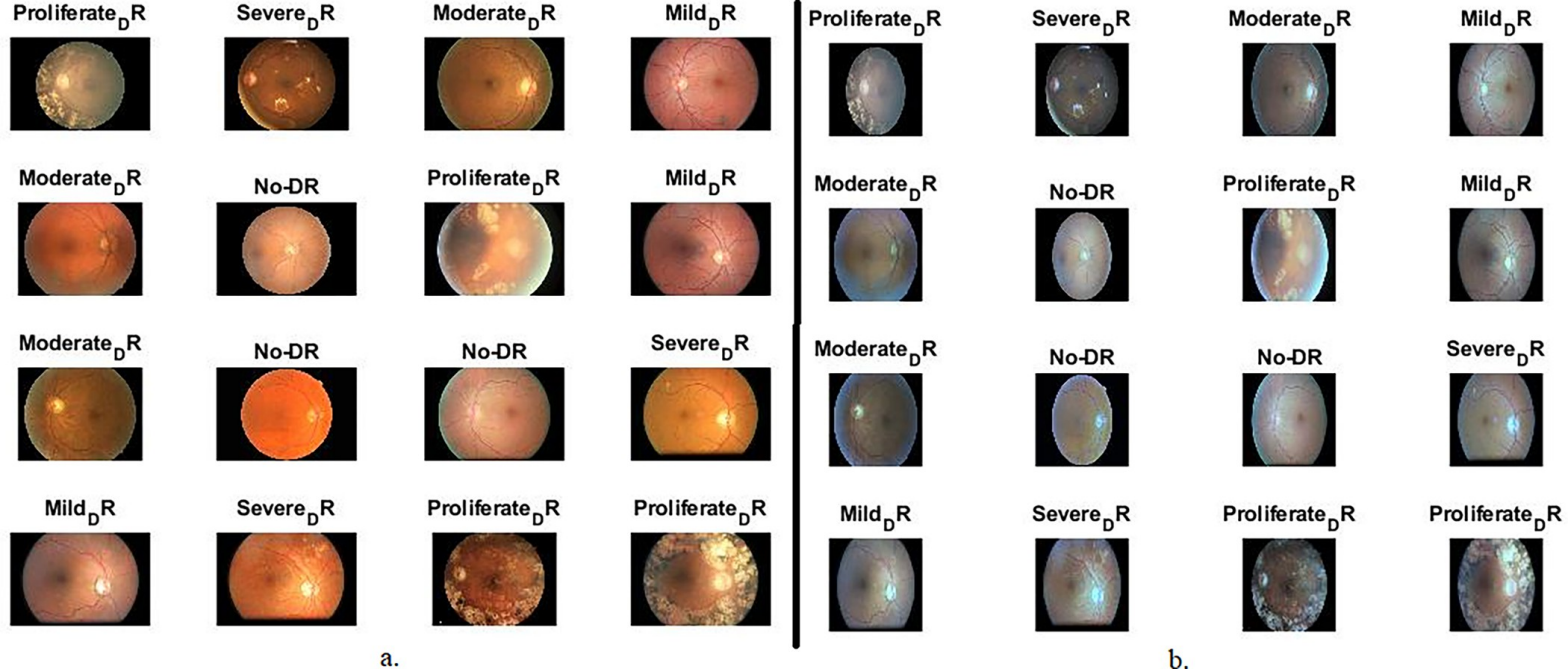
Image samples of the DR data set a. before improving b. after improving.

In this work, training and testing of systems performance have been conducted on the DR data set [22]. The dataset contains five stages of DR development with a total of 35,126 retinal fundus images. The images were collected from different types of cameras from different devices and this negatively affects the performance of the systems. All fundus images with the size of 3500 x 3000 pixels on 24-bit RGB colour channels. The dataset images are divided among 5 DR stages as follows: 25810 retinal colour fundus images of class No_DR, 2443 images of class diabetic retinal Mild_DR, 5292 images of diabetic retinal of class Moderate_DR, 873 images of diabetic retinal of class Severe_DR, and 708 images of diabetic retinal of class Proliferate_DR. It is noted that the No_DR class represents more than 73% of the data set and the accuracy will tend towards this class. Therefore, 3097 diabetic retina images, by 12% of the Class No_DR images, have been randomly selected. Thus, the proposed systems were trained and tested on a data set of the DR with a size of 12413 images distributed, as shown in Table 2. Table 1 describes the most critical characteristics and biomarkers for each stage of DR development. Fig 3A shows 16 random images from the data set for each stage.

**Table 1:** Interpretation of characteristics and biomarkers of DR patients during all stages.

DR Stages	لBiomarkers during each stage
Not_DR	Normal images of diabetics without any abnormalities
Mild_DR	The appearance of signs of a microaneurysm slightly
Moderate_DR	The appearance of signs of microaneurysm greater than Mild_DR stage
	The appearance of soft secretions with the shape of white circular
Severe_DR	The appearance of soft and hard secretions of white and yellow colour, circular and irregular in shape
	The bleeding appears in a quarter or more of the eye
	The appearance of severe abnormalities of the small blood vessels in a quarter or more of the eye
	The prominence of tiny blood vessels in a quarter or more of the eye
Proliferative_DR	Excessive pre-retinal hemorrhage
	New abnormal blood vessels appear

### 3.2. Enhancing the DR images

The presence of artifacts and low contrast are challenges for image processing and deep learning models. Therefore, this challenge must be addressed as the first step in biomedical image processing. The DR images contain artifacts and low contrast due to eyelashes, eye movement, and different fundus cameras. Therefore, these things represent a challenge and lead to unsatisfactory diagnostic results. In this study, the average colours of the RGB channels were calculated. The scaling of the DR images was also adjusted to calculate the scaling of the colour of the DR images.

An average filter is applied to remove artifacts. First, the filter is set to 5 * 5 and starts working; each time, it chooses a target pixel, takes 24 adjacent pixels and calculates the average, then changes the target pixel by the average values [23]. The filter continues and, on each iteration, shifts the target pixel by the adjacent average according to Eq 1.

$$Z(n)=\frac{1}{M}\sum_{j=0}^{M-1}S(n-i)$$ 1

where *Z*(*n*) is the input, *S*(*n*−i) is the previous input, and M is number pixels.

Some images have low contrast between the small blood vessels and the surrounding regions and low contrast between the white and yellow secretions with the surrounding regions. Therefore, the Contrast limited adaptive histogram equalization (CLAHE) method was applied to show the low variance [24]. The method works by spreading the bright pixels on the dark areas, which improves the appearance of the edges of the small blood vessels and the white and yellow spots. The method works by selecting a pixel and comparing it with its neighbour and making a decision according to this comparison. When the selected pixel is larger than its neighbours, the contrast increases, while when the selected pixel is smaller than its neighbours, the contrast decreases. The method continues until each pixel is compared to its neighbours, eventually leading to improved DR images and increased contrast of ROI. Fig 3B shows 16 random images from the RD data set for each stage after optimization.

### 3.3. ANN with features of CNN models

In this section, a technique between CNN and ANN has been applied to predict the stages of DR. The reasons for using hybrid techniques are that they require a medium-cost computer, faster data set training, and better results than CNN models. The process extracts feature from the DR images and reduce their dimensions by the PCA method, then classified by ANN.

#### 3.3.1. CNN of features extracting

CNN models have superior feature extraction capabilities without manual intervention. CNN models have many layers with millions of parameters and connections, which distinguishes them from machine learning methods. This work focuses on extracting features by DenseNet-121 and AlexNet models from the DR dataset. Each image of the DR dataset passes through many convolutional, auxiliary and pooling layers. Each layer contains millions of neurons and undergoes computations to perform a particular task [25].

Convolutional layers in CNN models whose number varies from one model to another, and their task varies from one layer to another. The main task of this layer is to extract the features, and each layer has a specific performance to extract particular features, such as the features of shape, geometric, colour, edges, and others. Some parameters that control the convolutional layer to help it extract the features are as follows: Filter size f (t) to wrap around the image based on selecting specific pixels from the image x (t) each time as in Eq 2. p-step, which determines how much the filter jumps on the image in each iteration [26]. Zero-padding Fills the edges of the processed image with zeros to maintain the same size as the original image.

W(t)=(x*f)(t)=∫x(a)f(t−a)da 2

W (t) refer to the output layer, f (t) refers to the filter and x (t) refer to the inputted DR image.

Convolutional layers are challenging because they produce millions of neurons, so CNN models solve this challenge through pooling layers. Pooling layers represent a group of pixels with a single pixel [27]. The max pooling layers represent a set of pixels and change them with the max value of the pixels as in Eq 3. The average pooling layers work on selecting a set of pixels of the processed image, calculating the average of the pixels and changing the set of pixels with their average value as in Eq 4.

$$z(i;\;j)={max}_{m,n=1\ldots .k}f[(i-1)p+m;\;(j-1)p+n]$$ 3

$$z(i;\;j)=\frac{1}{k^{2}}\sum_{m,n=1\ldots .k}f[(i-1)p+m;\;(j-1)p+n]$$ 4

where f refers to the filter size, m, n refers to the matrix location, p refers to the moving filter, and k is the vectors.

Finally, each model produces high-dimensional features with a size of 12413 x 2048. The PCA method was applied to reduce the dimensions, select the most critical features, and save them at a size of 12413 x 512 for each model.

#### 3.3.2. The ANN classifier

ANN is a highly efficient neural network for classification tasks. An ANN network consists of three basic layers. First, the input layer, through which the network is fed with features extracted from the previous stage (features of CNN models) [28]. In this study, the input layer contains 12413 units. Second, hidden layers in which complex mathematical operations are performed to perform their tasks. The hidden layers consist of 12 hidden layers connected between them by weight connections. The output layer represents the network’s output after performing the required tasks. The output layer consists of 5 layers; each layer represents one class of RD dataset. In each iteration, the Mean Squared Error (MSE) is calculated by the difference between the expected values *Y*_*j*_ and the actual *Z*_*j*_, as in Eq 5. The weights continue to be updated until the network reaches the MSE.

$$MSE=\frac{1}{M}\sum_{j=1}^{M}\;{\left(Y_{j}-Z_{j}\right)}^{2}$$ 5

where M refer to the data points, *Y*_*j*_ refer to the actual value, and *Z*_*j*_ refer to the expected value.

**Fig 4:**
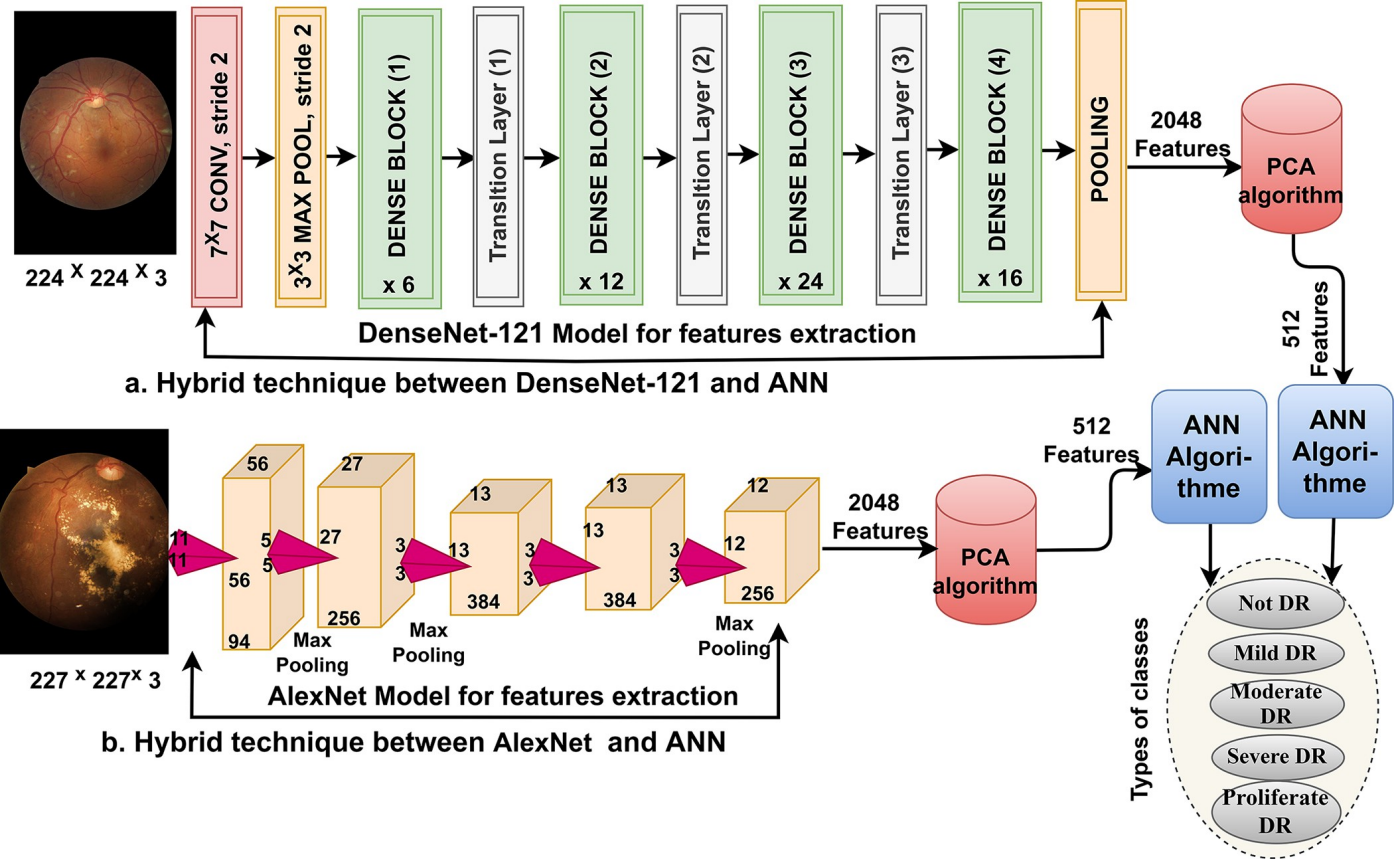
The framework of the hybrid technique for diagnosing DR dataset by ANN with CNN features.

Fig 4 shows the architecture of the hybrid technique with low-dimensional CNN features for diagnosing the DR dataset.

### 3.4. ANN classifier based on integrating features of CNN models

**Fig 5:**
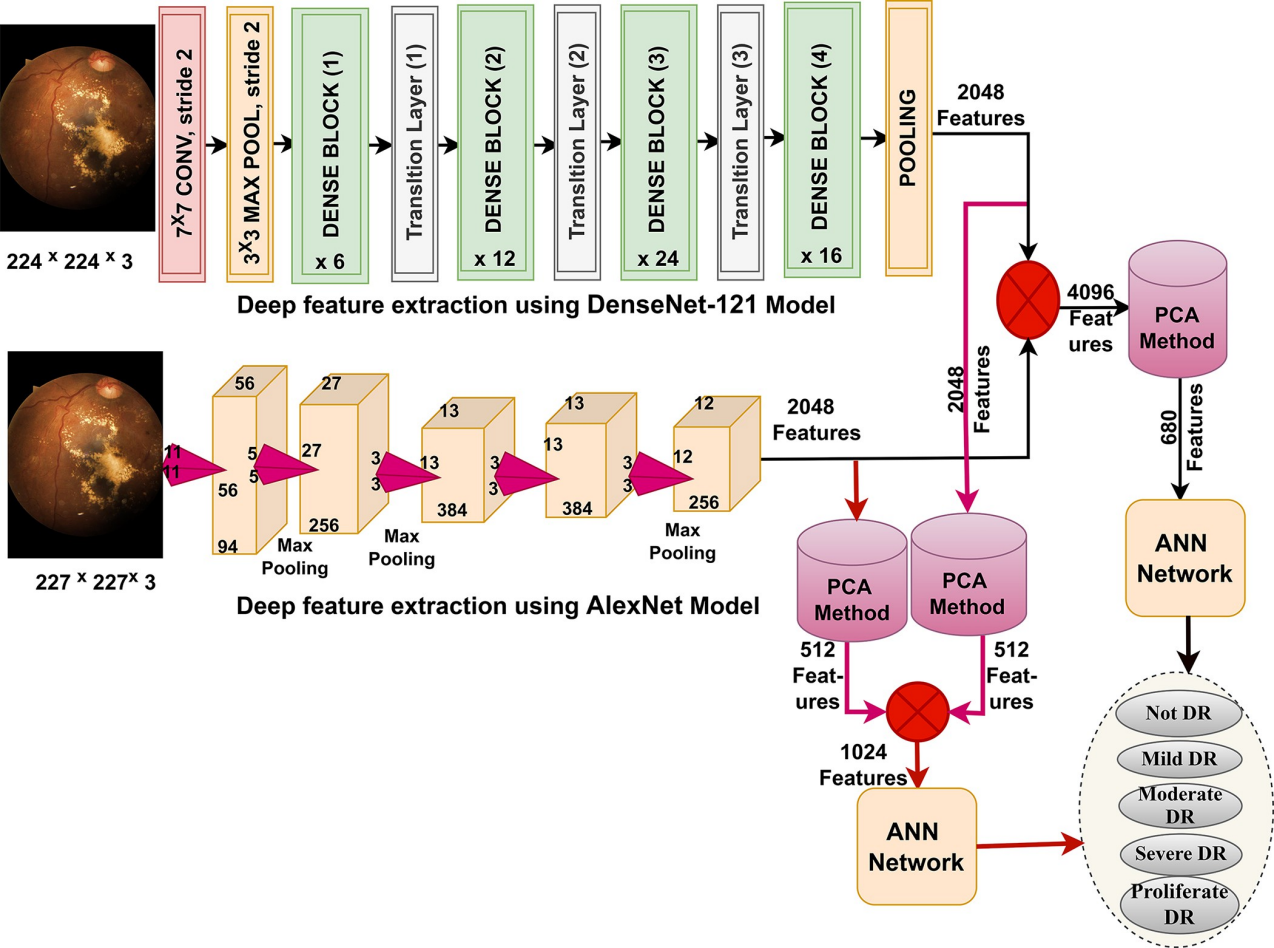
The framework of diagnosing DR dataset through the hybrid features.

This section presents the diagnosis of the DR dataset by ANN classifier when fed with hybrid features of Dense-121 and AlexNet models. The rationale for applying this technique is the speed of training and testing of the data set and its accurate results to distinguish between the stages of DR. Fig 5 shows the structure of two systems for diagnosing the DR dataset.

The first system has been passed through several processes as follows: First, the imagery of the DR dataset is optimized and fed into the Dense-121 and Alex models. Second, the Dense-121 and Alex models analyze the DR images, extract the features, and save them in vectors with a size of 12413 x 2048 for each model. Third, the vectors of both models are combined into new vectors of size 12413 x 4096. Fourth, because of the high features, PCA is applied to reduce dimensions and select important features and save them in new vectors size of 12413 x 680. Fifth, to classify the hybrid features, the ANN classifier is fed by vectors size of 12413 x 680. Finally, ANN trains the hybrid features of DR and tests ANN performance.

The second system has been passed through many processes: The first and second processes are the same as the processes of the first system. Third, because of the high features of the Dense-121 model, PCA is applied to reduce the dimensions, select essential features, and save them in new vectors of size 12413 × 512. Fourth, because of the high features of the Alex model, PCA is applied to reduce the dimensions, select essential features, and save them in new vectors of size 12413 x 512. Fifth, the vectors of both models are combined into new vectors of size 12413 x 1024. Sixth, to classify the hybrid features, the ANN classifier is fed by vectors size of 12413 x 1024. Finally, ANN trains the hybrid features of DR and tests ANN performance.

### 3.5. ANN classifier based on integrating the features of CNN and handcrafted features

**Fig 6:**
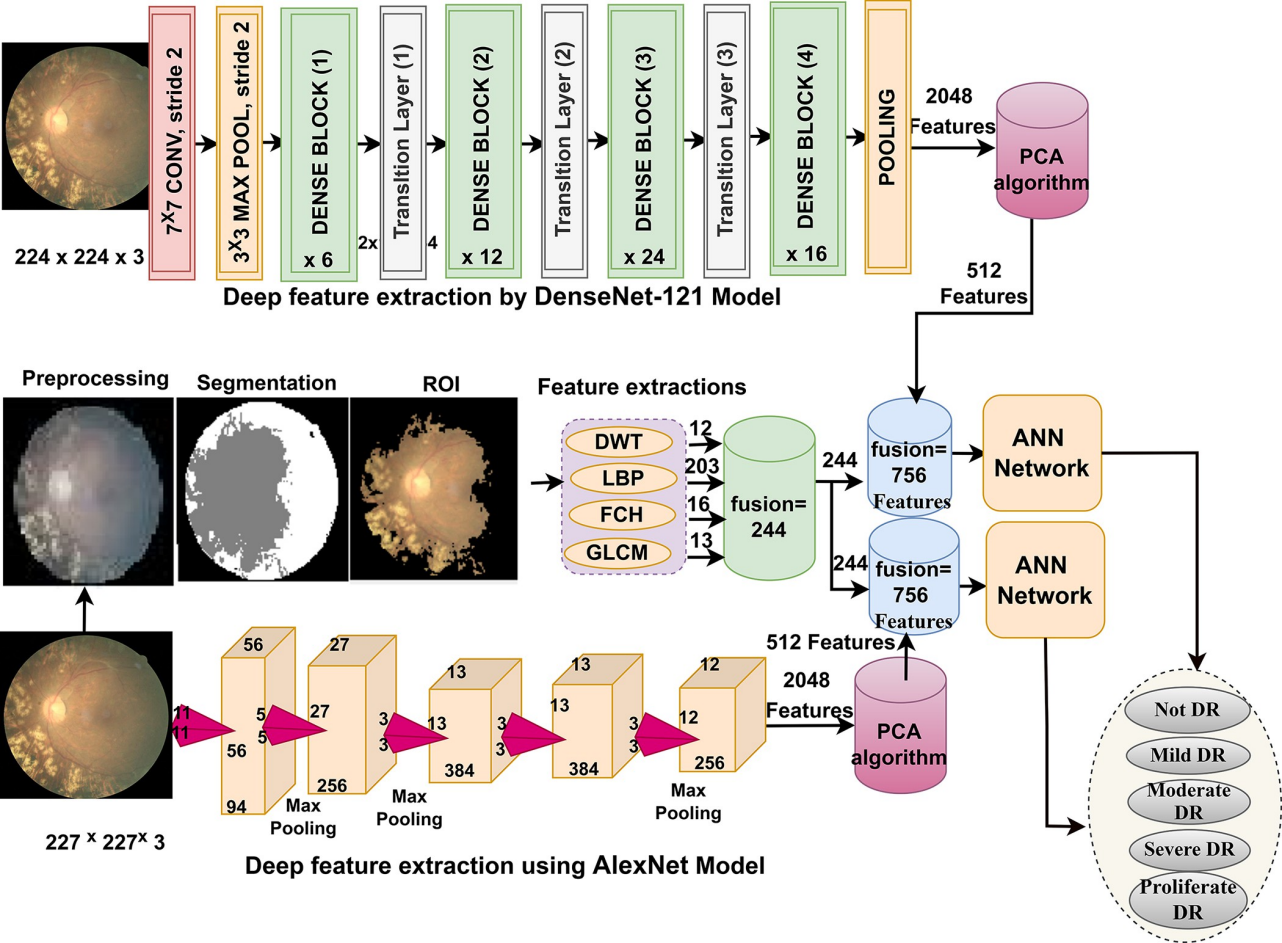
The framework of the structure of the hybrid method with radiomic features.

Here is a novelty methodology for fundus image characterization of the DR dataset by ANN classifier with integrative features between CNN (Dense-121 and Alex) models and features of DWT, LBP, FCH, and GLCM methods (handcrafted features) [29]. Fig 6 illustrates the framework of the hybrid technique with hybrid features for diagnosing colour fundus images of the DR dataset, which consists of two systems.

The second system has been passed through many processes: First, the imagery of the DR dataset is optimized and fed into the Dense-121 and Alex models. Second, the Dense-121 and Alex models analyze the DR images, extract the features, and save them in vectors with a size of 12413 x 2048 for each model. Third, because of the high features, PCA is applied to reduce dimensions and select important features and save them in new vectors size of 12413 x 512 for the Dense-121 model and 12413 x 512 for the Alex model. Fourth, the improved colour fundus images of the DR dataset are passed to the DWT, LBP, FCH, and GLCM methods, each of which produces special features as follows.

The first algorithm to extract the characteristics of fundus images is DWT, which divides the image into four parts (components). It applies a specific filter to each part of the image. Each filter produces three statistical features from each section; thus, 12 features are created from the whole image. The low filter is passed on the first section of the image to analyze the approximate parameters and produce the statistical features are the standard deviation, the mean, and the variance. Low-High and High-Low filters are passed on an image’s third and fourth sections to analyze the detailed parameters and produce three statistical features from each section. The high filter is passed in the fourth section of the image to analyze the detailed parameters and create three statistical features. Finally, the algorithm produces 12 features and saves them to vectors of size 12413 x 12.

The second algorithm for extracting features from fundus images is LBP, which extracts surface texture features by converting the retinal fundus image into a gray system and representing it in a matrix [30]. The algorithm extracts spatial information through local contrast. The algorithm is set to 4 * 4 which means a central pixel *g*_*c*_ and 15 adjacent *g*_*p*_. Each time each pixel is changed according to the algorithm’s mechanism of 14 adjacent pixels as in Eq 6. Finally, the algorithm produces 203 features and saves them in vectors of size 12413 x 203.

$${LBP}_{R,P}=\sum_{p=0}^{P-1}s(g_{p}-g_{c})2^{p}$$ 6

where *g*_*c*_ refers to the aim pixel, *g*_*p*_ refers to adjacent pixels R is the adjacent radius, and P is the number of adjacent pixels.

Third algorithm for extracting features from fundus images is FCH which extracts colour features from fundus images with the fuzzy logic system. The colour features are one of the best features for distinguishing the DR stages [31]. The colours of the ROI are represented in the histogram bin. Any colours in one histogram bin are the same even if the colours are different. Each histogram bin has different colours. The colours of the ROI are thus represented in fuzzy logic containers. Finally, the algorithm produces 16 features and saves them in vectors of size 12413 x 16.

The fourth algorithm for extracting features from fundus images is GLCM, which extracts texture features by converting the image to grayscale and representing it in a matrix [32]. The algorithm analyzes spatial information to extract features from ROI. The algorithm extracts the features based on a comparison of pixels with neighbours based on distance d and principal angles 0°, 45°, 90° and 135°. The algorithm decomposes the image regions into smooth and coarse; Smooth regions have very close or equal pixels, while coarse regions have very uneven pixels. Finally, the algorithm produces 13 features and saves them in vectors of size 12413 x 13.

Fifthly, the features produced by the four algorithms called handcrafted features are combined in vectors of size 12413×244.

Sixth, the low-dimensionality of the Dense-121 model is combined with the handcrafted features in vectors size of 12413 x 756 are which called radiomic features

Seventh, the low-dimensional Alex model features were combined with the handcrafted features in vectors size of 12413 x 756 which are called radiomic features.

## 4. The results of systems

### 4.1 Split of DR dataset

Several systems were applied in this work to diagnose and differentiate between stages of progression of diabetic fundus images of the DR dataset. The dataset contains 12413 images distributed unequally between five stages of DR progression. The colour fundus images of the DR data set are distributed among the stages (classes) of its development as follows: 3097 retinal images of class No_DR with a rate of 25%, 2443 images of DR of class Mild_DR at a rate of 19.68% and 5292 images of the diabetic retina of class Moderate_DR with a rate of 42.6% and 873 diabetic retinal images of class Severe_DR with a rate of 7.02%, and 708 images of diabetic retinal of class Severe_DR with a rate of 5.7%. Table 2 shows the division of the data set to 80% during the training phase of systems on the DR data set and its validation by (80:20) and during the evaluation of the system’s performance by 20%.

**Table 2:** Splitting of the DR images through all stages.

Phase	80% from dataset		Testing (20%)
Classes	Training (80%)	validation (20%)	
Not_DR	1982	496	619
Mild_DR	1563	391	489
Moderate_DR	3387	847	1058
Severe_DR	558	140	175
Proliferative_DR	453	113	142

### 4.2 Evaluation metrics

All systems were evaluated on the colour fundus images of the DR dataset by several scales indicated by Eqs 7–11. Each system generates the confusion matrix as the best system evaluation tool, which represents correctly (TN and TP) and incorrectly (FN and FP) classified images. Thus, the equations take their data from the confusion matrix [33].


$$Sensitivity=\frac{TP}{TP+FN}*100\%$$ 7

$$AUC=\frac{TP\;Rate}{FP\;Rate}$$ 8

$$Accuracy=\frac{TN+TP}{TN+TP+FN+FP}*100\%$$ 9

$$Specificity=\frac{TN}{TN+FP}*100$$ 10

$$Precision=\frac{TP}{TP+FP}*100\%$$ 11

### 4.3. Balancing dataset and data augmentation

The small number of medical images is insufficient to train CNN models, which is one of the limitations that cause the overfitting of CNN models. Another challenge facing CNN models is the imbalanced classes in the data set. Thus, these two challenges were overcome by applying the data augmentation method. First, this method increases the data set’s images to overcome the overfitting problem. Data augmentation is a technique commonly used in machine learning and computer vision tasks to increase the size and diversity of a training dataset. In the context of image analysis, data augmentation involves applying various transformations or modifications to existing images to create new training samples. The primary purpose of data augmentation is to provide additional variations of the original images, which helps the model generalize better to real-world scenarios and improve its overall performance. By augmenting the data, we can increase the number of training samples without the need for collecting or labeling new images, which can be time-consuming and expensive. Data augmentation techniques typically involve applying a combination of geometric and color transformations to the original images. In this work, the augmentedImageDatastore function has been used, which increases the number of images from the same dataset by several operations as follows: Some commonly used transformations include: Horizontal or vertical flipping: The image is flipped horizontally or vertically, creating a mirror image. This augmentation is useful when the orientation or symmetry of objects in the image is not significant. Rotation: The image is rotated by a certain angle, introducing variations in object orientations. This augmentation is particularly helpful when the orientation of objects is essential for classification or detection tasks. Scaling and cropping: The image is resized to different scales or cropped to focus on specific regions of interest. This augmentation allows the model to learn robustness to variations in object sizes or positions. Translation: The image is shifted horizontally or vertically, simulating different object placements within the image. This augmentation helps the model become invariant to slight shifts in object positions. Brightness and contrast adjustment: The brightness or contrast of the image is modified, creating variations in lighting conditions. This augmentation helps the model become more robust to changes in illumination. By applying these transformations to the existing images in the dataset, we can generate multiple augmented versions of each image, effectively increasing the size and diversity of the training dataset. This expanded dataset exposes the model to a wider range of variations, making it more robust and capable of generalizing well to unseen data during the training process.

**Fig 7:**
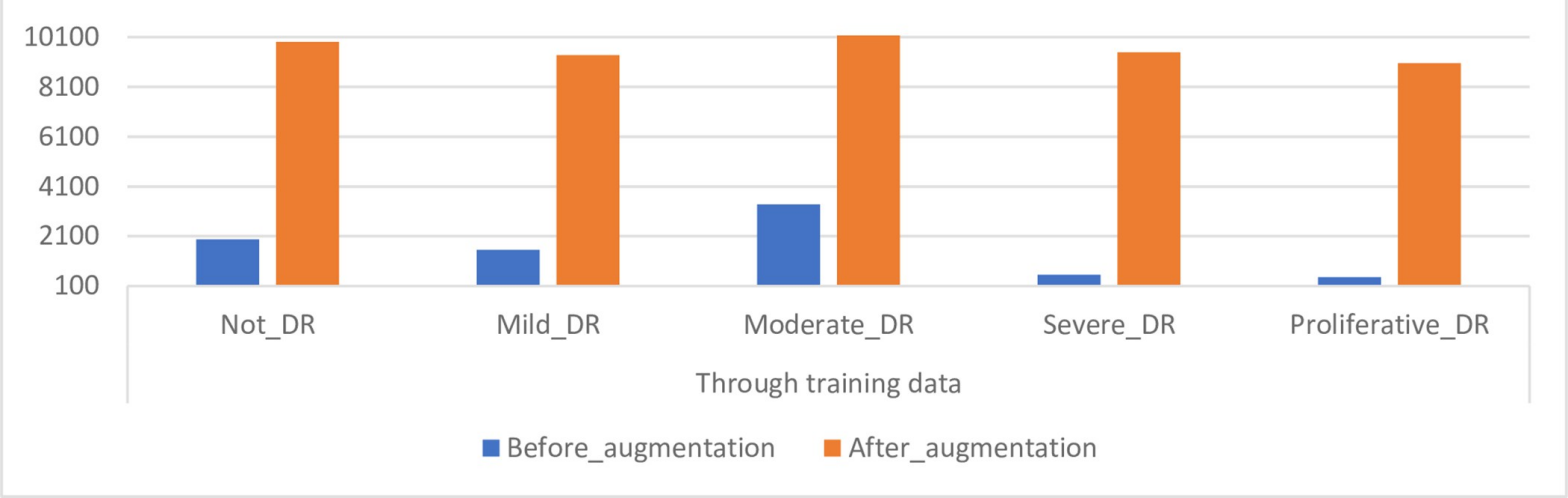
Classes distribution of the DR data set before and after balancing.

Secondly, the method balances the data set classes by increasing the pictures in each class differently [34]. It increases the images of the majority classes less than the minority classes [35]. Thus, we get classes balanced data set. Fig 7 shows the distribution of classes for the DR data set before and after the data augmentation was applied. Notes the importance of this method in balancing the data set. Table 3 summarizes the number of DR data set images during each phase (class) before and after data augmentation during the training of DR data set images.

**Table 3:** Balancing classes of DR data set during training of a data set.

Phase	Through training data				
Stages name	Not_DR	Mild_DR	Moderate_DR	Severe_DR	Proliferative_DR
Before_aug	1982	1563	3387	558	453
After_aug	**9,910**	**9,378**	**10,161**	**9,486**	**9,060**

### 4.4. Results of ANN with features of CNN models

This section discusses a summary of the results of the performance of the ANN with the features of both Dense-12 and Alex after reducing the high dimensionality of the features of the colour fundus images of the DR dataset. This method extracts the features of the DR images from the Dense-12 and Alex models separately. The most critical features have been selected, reduce the high dimensions by PCA, and save the essential features for each model separately. Important and low-dimensional features are fed to the ANN classifier, which distributes them during the training and testing phases.

**Fig 8:**
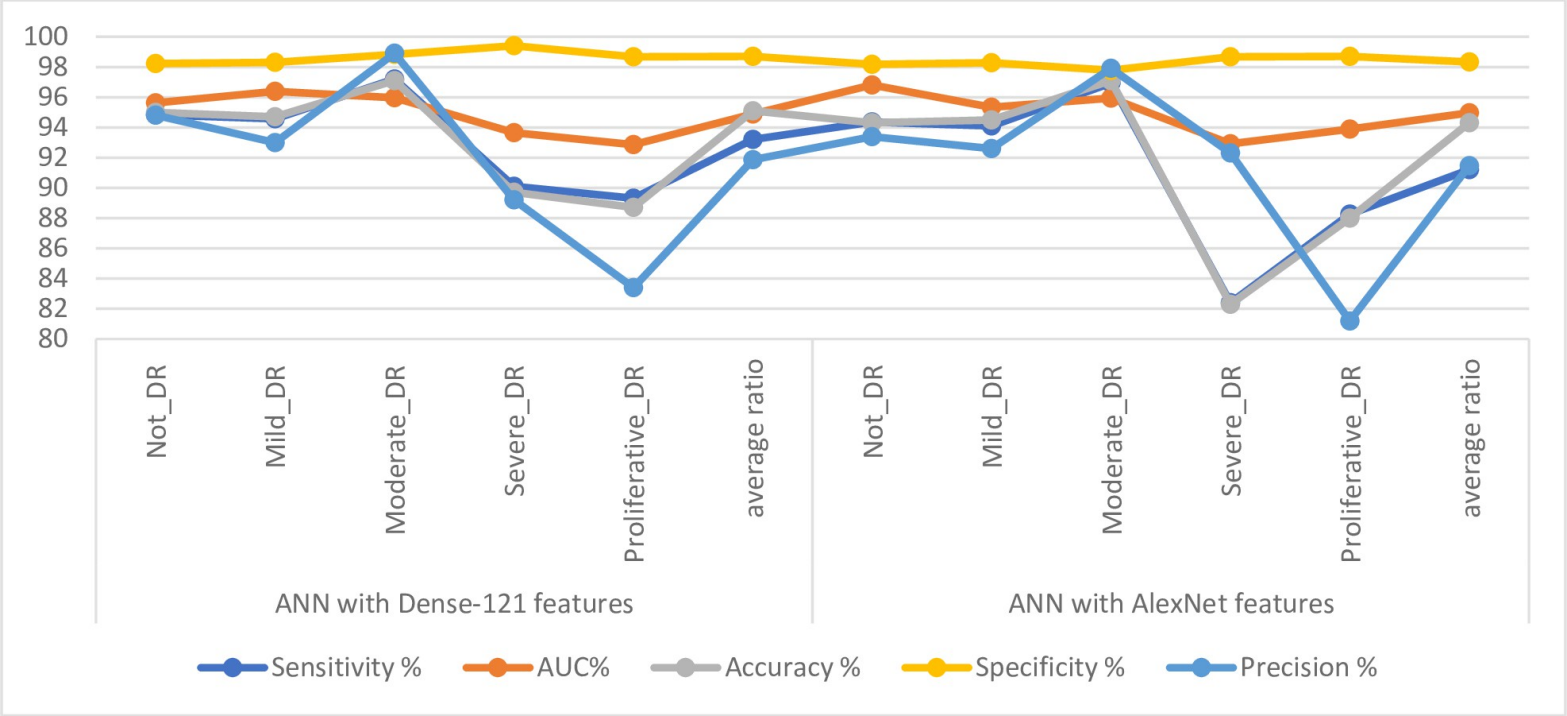
Displaying the results of DR image diagnostics by ANN with Dense-121and Alex features for detection of DR development.

Table 4 and Fig 8 discuss the summary of the results of the ANN classifier with the significant and low-dimensional features of the Dense-121and Alex models. ANN with the low-dimensional features of the Dense-121 obtained better results than it did with the low-dimensional features of the Alex model. With Dense-121 features, ANN has achieved a sensitivity of 93.20%, an AUC of 94.89%, an accuracy of 95.1%, a specificity of 98.69% and a precision of 91.86%. In contrast, with features of Alex, the ANN has achieved a sensitivity of 91.21%, an AUC of 94.97%, an accuracy of 94.3%, a specificity of 98.32% and a precision of 91.48%.

**Table 4:** Summary of ANN performance with Dense-121 and AlexNet features.

Techniques	Type of Class	Sensitivity %	AUC%	Accuracy %	Specificity %	Precision %
ANN with Dense-121 features	Not_DR	94.84	95.62	95.00	98.22	94.80
	Mild_DR	94.56	96.38	94.70	98.30	93.00
	Moderate_DR	97.2	95.96	97.10	98.84	98.90
	Severe_DR	90.1	93.64	89.70	99.40	89.20
	Proliferative_DR	89.32	92.86	88.70	98.68	83.40
	**average ratio**	**93.20**	**94.89**	**95.10**	**98.69**	**91.86**
ANN with AlexNet features	Not_DR	94.36	96.81	94.30	98.17	93.40
	Mild_DR	94.11	95.32	94.50	98.28	92.60
	Moderate_DR	96.94	95.94	97.10	97.78	97.90
	Severe_DR	82.40	92.92	82.30	98.68	92.30
	Proliferative_DR	88.25	93.88	88.00	98.70	81.20
	**average ratio**	**91.21**	**94.97**	**94.30**	**98.32**	**91.48**

**Fig 9:**
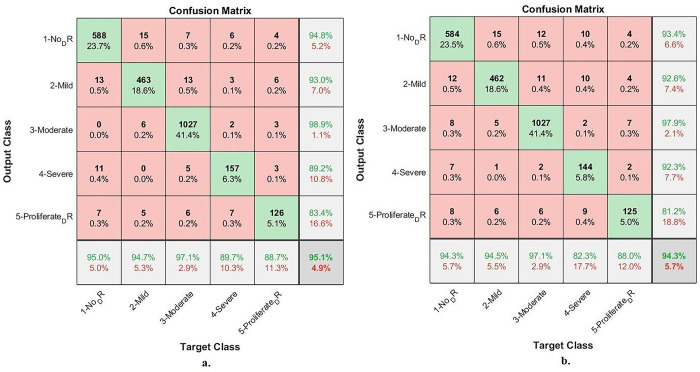
ANN performance for early-stages DR dataset image diagnostics with features of a. Dense-121 b. Alex.

Fig 9 summarizes the assessment of the ANN through the confusion matrix to detect the stages of DR development before blinding. With Dense-121 features, ANN has achieved an accuracy of 95.1% and accuracy for each class as follows: Not_DR of 95%, Mild_DR of 94.7%, Moderate_DR of 97.1%, Severe_DR of 89.7%, and Proliferative_DR of 88.75%. In contrast, with Alex features, ANN has achieved an accuracy of 94.3% and accuracy for each class as follows: Not_DR of 94.3%, Mild_DR of 94.5%, Moderate_DR of 97.1%, Severe_DR of 82.3%, and Proliferative_DR of 88%.

### 4.5. Results of ANN classifier based on integrating features of CNN models

This section discusses a summary of ANN performance results with combined Dense-12 and Alex features for image diagnosis of the DR dataset before it progresses to critical stages. The technology in this method consists of two systems; each system has a working mechanism as follows: The first system extracts the features of Dense-12 and Alex models and combines them into the same vectors; then feeds the combined vectors to the PCA to select the essential features and reduce their high dimensions. Low-dimensional important feature vectors are fed to the ANN classifier for distribution during the system’s training and performance testing phases. In the second system in which, the features of the Dense-12 model are extracted; then fed the vectors to the PCA to select the essential features and reduce their high dimensions. Then extracted Alex model features and fed them to the PCA method to select important features and reduce their high dimensions. After reducing the dimensions of both Dense-12 and Alex models, they are combined into the same vectors. The vectors that contain the essential features are fed to the ANN classifier for distribution during the system’s training and performance testing phase.

**Fig 10:**
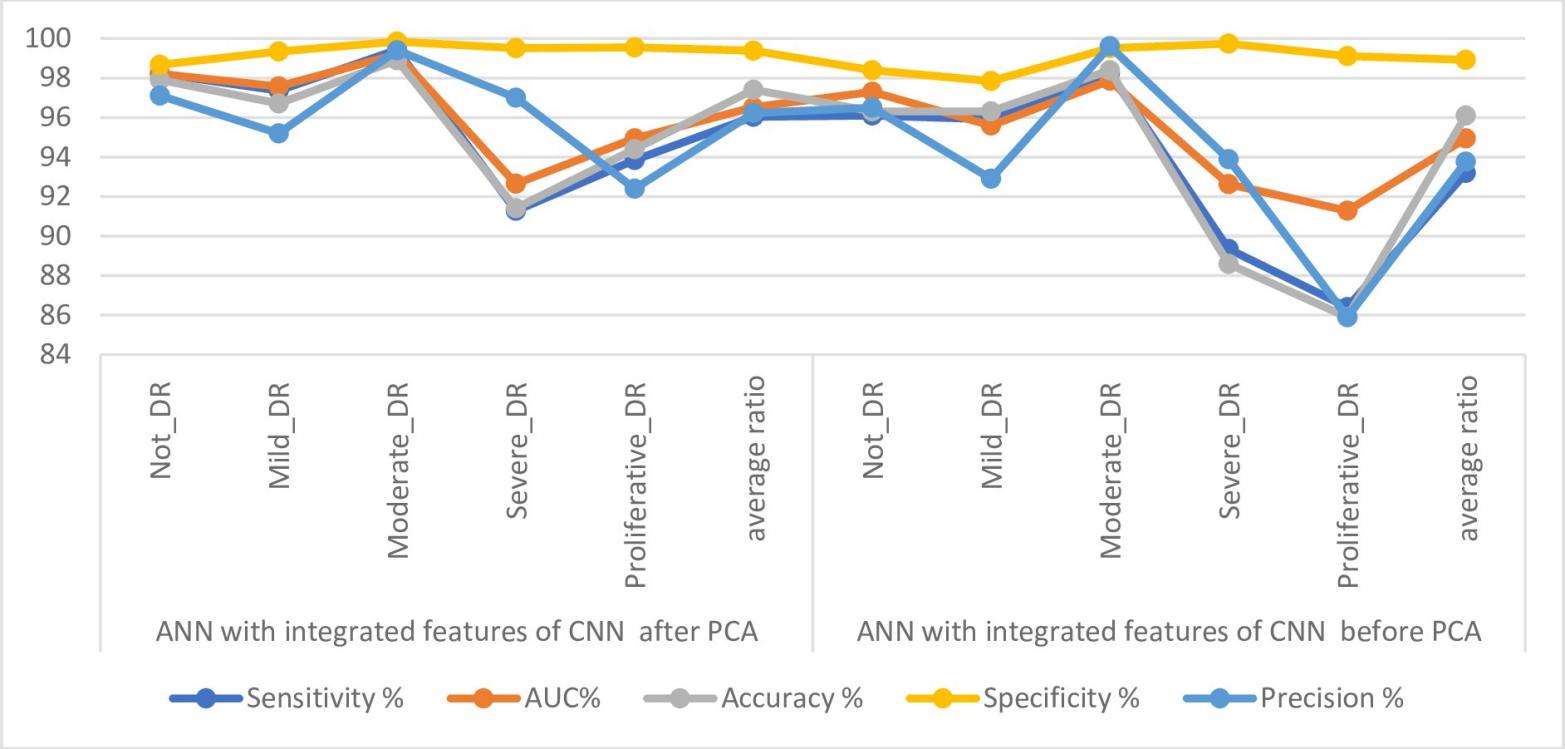
Displaying the results of DR image diagnostics by ANN with integrated features of CNN for detection of DR development.

Table 5 and Fig 10 discuss the summary of the results of the ANN classifier with the essential features combined before PCA and after PCA of the Dense-121 and Alex models. ANN with combined features of Dense-12 and Alex after PCA got better results than combining features before PCA. With the combined features of after PCA, ANN has achieved a sensitivity of 96.03%, an AUC of 96.51%, an accuracy of 97.4%, a specificity of 99.38% and a precision of 96.22%. In contrast, with features combined before PCA, ANN achieved a sensitivity of 93.20%, an AUC of 94.93%, an accuracy of 96.1%, a specificity of 98.92% and a precision of 93.76%.

**Table 5:** Results of ANN with integrated features of CNN.

Techniques	Classes of DR	Sensitivity %	AUC%	Accuracy %	Specificity %	Precision %
ANN with integrated features of CNN after PCA	Not_DR	98.23	98.20	97.90	98.67	97.10
	Mild_DR	97.36	97.58	96.70	99.34	95.20
	Moderate_DR	99.42	99.20	98.90	99.84	99.40
	Severe_DR	91.28	92.64	91.40	99.50	97.00
	Proliferative_DR	93.85	94.95	94.40	99.55	92.40
	**average ratio**	**96.03**	**96.51**	**97.40**	**99.38**	**96.22**
ANN with integrated features of CNN before PCA	Not_DR	96.10	97.30	96.30	98.40	96.50
	Mild_DR	95.90	95.60	96.30	97.84	92.90
	Moderate_DR	98.24	97.87	98.40	99.50	99.60
	Severe_DR	89.35	92.62	88.60	99.74	93.90
	Proliferative_DR	86.40	91.28	85.90	99.10	85.90
	**average ratio**	**93.20**	**94.93**	**96.10**	**98.92**	**93.76**

**Fig 11:**
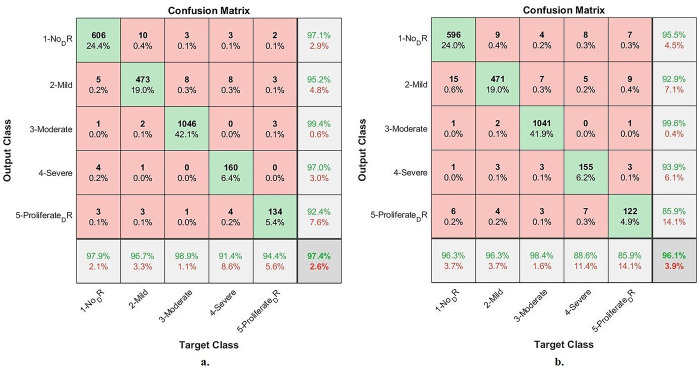
ANN performance for early-stages DR dataset image diagnostics with combined features of Dense-121 and Alex a. after PCA b. before PCA.

Fig 11 summarizes the assessment of the ANN through the confusion matrix to detect the stages of DR development before blinding. With the combined features after PCA, ANN has achieved up to 97.4% accuracy and for each class, accuracy is as follows: Not_DR of 97.9%, Mild_DR of 96.7%, Moderate_DR of 98.9%, Severe_DR of 91.4%, and Proliferative_DR of 94.4%. In contrast, with the combined features before PCA, ANN has achieved an accuracy of 96.1% and an accuracy for each class as follows: Not_DR of 96.3%, Mild_DR of 96.3%, Moderate_DR of 98.4%, Severe_DR of 88.6%, and Proliferative_DR of 85.9%.

### 4.6. Results of ANN classifier based on radiomic features

This section discusses a summary of ANN performance results with radiomic features of CNN (Dense-12 and Alex) with handcrafted features (DWT, LBP, FCH, and GLCM) to diagnose colour fundus images of the DR dataset before they develop to critical stages. The technique in this method consists of two systems; the two systems differ in the combined features as follows: The first system for diagnosing colour fundus images of the DR dataset by ANN with combined features of Dense-12 and handcrafted features. The second system for diagnosing colour fundus images of the DR dataset by ANN with combined features of Alex and handcrafted features.

#### 4.6.1. Confusion matrix

**Fig 12:**
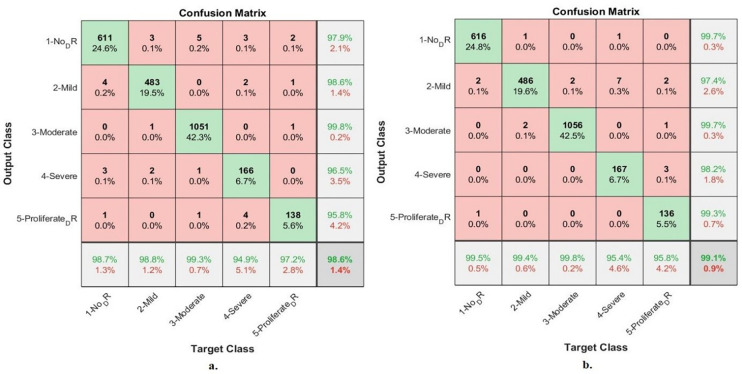
ANN performance for early-stages DR dataset image diagnostics with radiomic features a. Dense-121 and handcrafted b. Alex and handcrafted.

Fig 12 summarizes the assessment of an ANN with the radiomic features through the confusion matrix to detect stages of DR development before blinding. With combined features of the Dense-121 and handcrafted features, ANN has achieved an accuracy of up to 98.6% and for each category, the accuracy is as follows: Not_DR of 98.7%, Mild_DR of 98.8%, Moderate_DR of 99.3%, Severe_DR of 94.9%, and Proliferative_DR of 97.2%. In contrast, with combined features of the Alex and handcrafted features, ANN achieved an accuracy of 99.1% and an accuracy for each category as follows: Not_DR of 99.5%, Mild_DR of 99.4%, Moderate_DR of 99.8%, Severe_DR of 95.4%, and Proliferative_DR of 95.8%.

**Fig 13:**
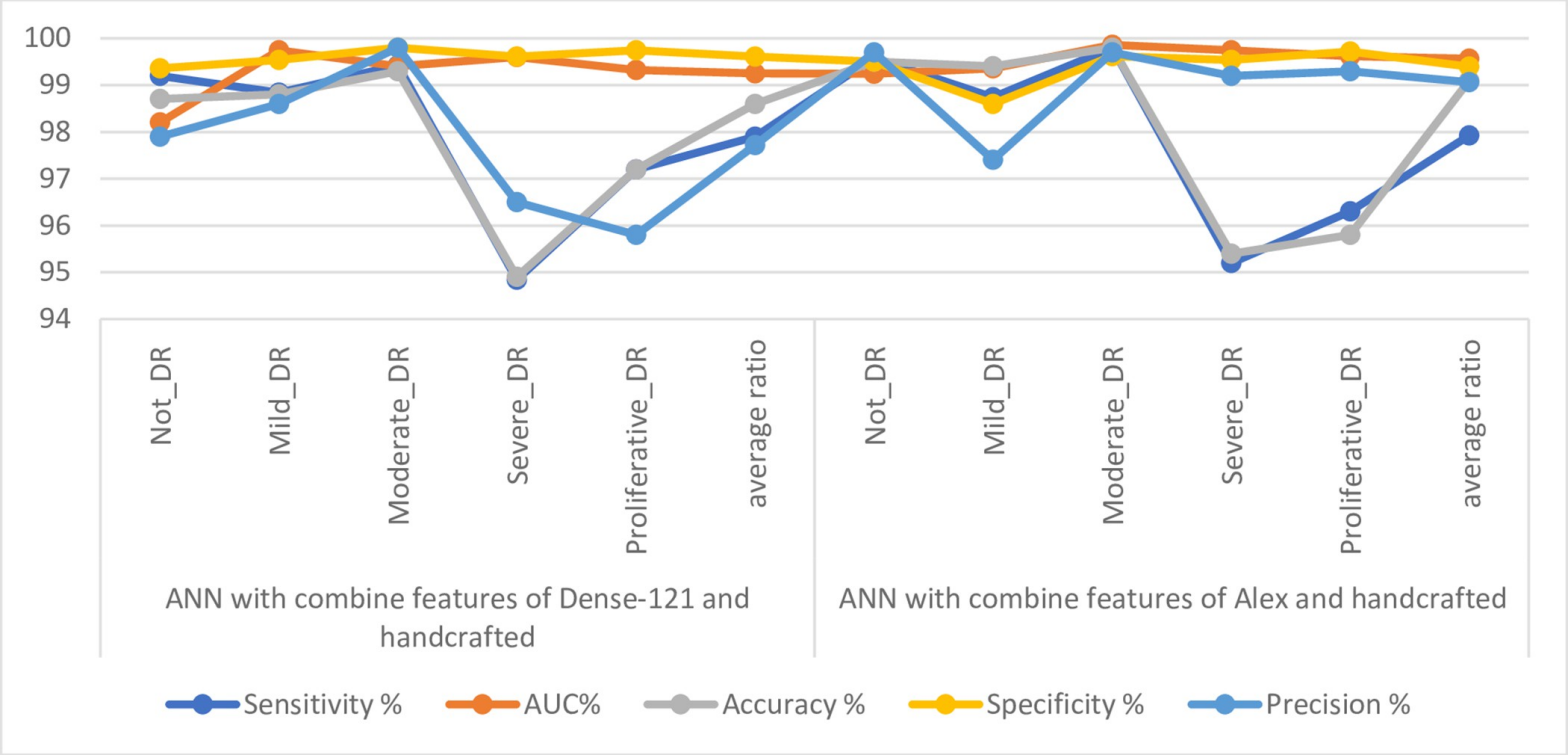
Displaying the results of DR image diagnostics by ANN with radiomic features for detection of DR development.

Table 6 and Fig 13 discuss the summary of the results of the ANN classifier with the hybrid radiomic features between the CNN models (Dense-12 and Alex) and the handcrafted features for image diagnostics of the early-stage DR dataset. With the combined features of Dense-121 and handcrafted features, the ANN achieved a sensitivity of 97.9%, an AUC of 99.25%, an accuracy of 98.6%, a specificity of 99.61% and a precision of 97.72%. In contrast, with combined features of Alex and handcrafted features, the ANN achieved a sensitivity of 97.92%, an AUC of 99.56%, an accuracy of 99.1%, a specificity of 99.4% and a precision of 99.06%.

**Table 6:** Results of ANN with radiomic features for diagnosing images of DR dataset.

Techniques	Classes of DR	Sensitivity %	AUC%	Accuracy %	Specificity %	Precision %
ANN with combine features of Dense-121 and handcrafted	Not_DR	99.2	98.20	98.70	99.36	97.90
	Mild_DR	98.84	99.74	98.80	99.54	98.60
	Moderate_DR	99.42	99.40	99.30	99.80	99.80
	Severe_DR	94.84	99.60	94.90	99.61	96.50
	Proliferative_DR	97.2	99.32	97.20	99.74	95.80
	**average ratio**	**97.90**	**99.25**	**98.60**	**99.61**	**97.72**
ANN with combine features of Alex and handcrafted	Not_DR	99.58	99.24	99.50	99.50	99.70
	Mild_DR	98.74	99.36	99.40	98.60	97.40
	Moderate_DR	99.80	99.86	99.80	99.62	99.70
	Severe_DR	95.20	99.74	95.40	99.54	99.20
	Proliferative_DR	96.30	99.62	95.80	99.72	99.30
	**average ratio**	**97.92**	**99.56**	**99.10**	**99.40**	**99.06**

#### 4.6.2. Cross-entropy

**Fig 14:**
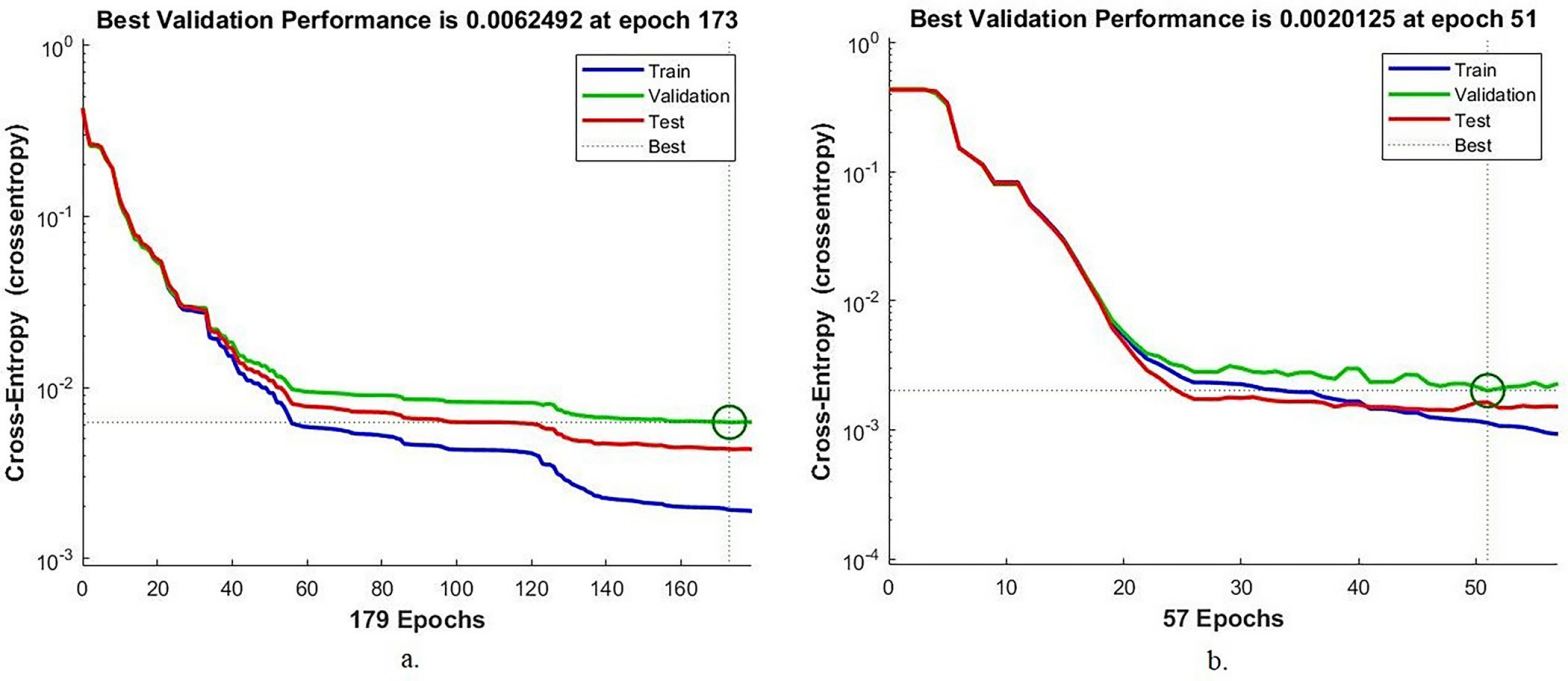
Display cross-entropy by ANN for early-stages DR dataset image diagnostics with radiomic features a. Dense-121 and handcrafted b. Alex and handcrafted.

Cross-entropy is one of the measurements that evaluate the performance of the ANN on the colour fundus images of the DR data set to diagnose its early stages before blindness. In each epoch, the network records the difference between the actual and expected values during each phase. Each stage has its own colour, as shown in Fig 14. The blue colour indicates the performance of the ANN on the DR images during the training of the data set. Green colour when adjusting the weights and parameters of the ANN network on the DR images. The red colour shows ANN’s performance on the DR images while testing new samples. With the combined features of Dense-121 and the handcrafted features, ANN achieved the lowest error by cross-entropy measure in the 173 epochs with a value of 0.0062492. In contrast, with Alex combined features and handcrafted features, ANN achieved the lowest error through the cross-entropy measure in the 51 epochs with a value of 0.0020125.

#### 4.6.3. Error histogram

**Fig 15:**
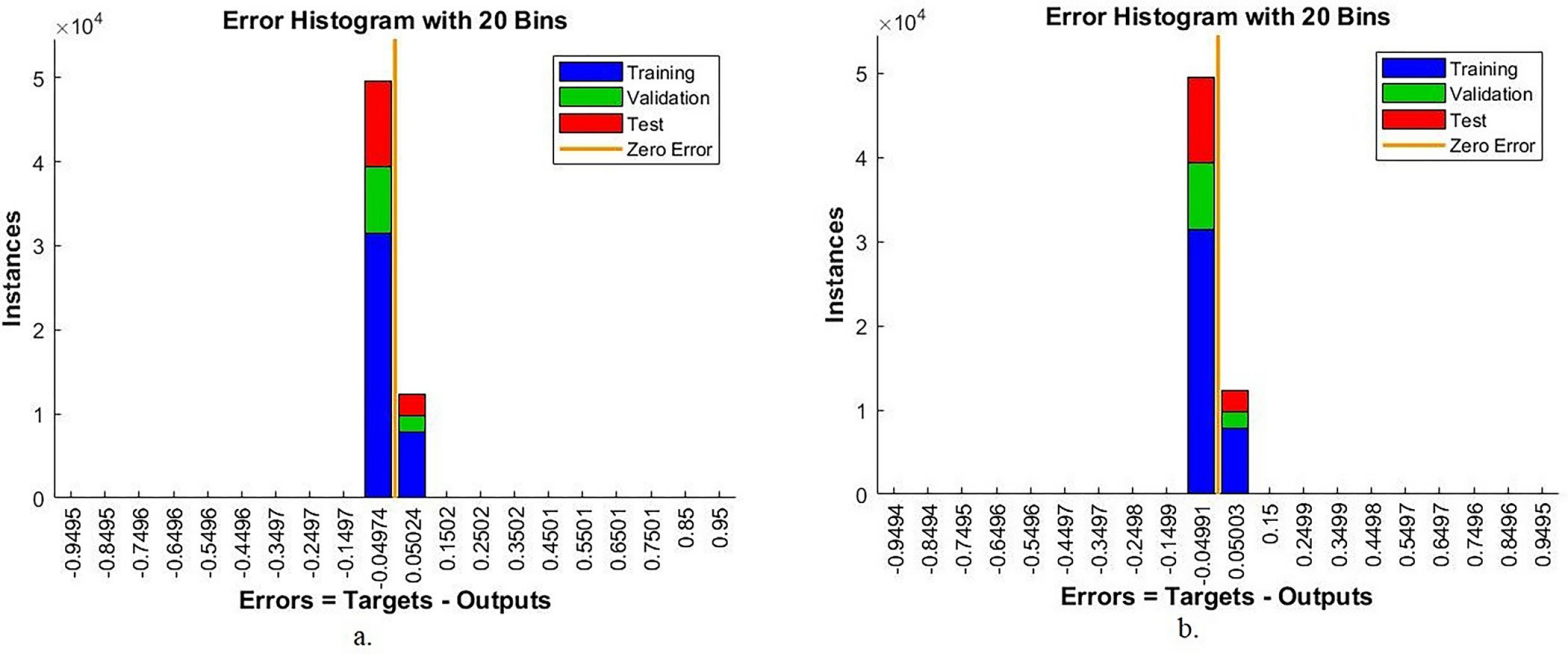
Display error histogram by ANN for early-stages DR dataset image diagnostics with radiomic features a. Dense-121 and handcrafted b. Alex and handcrafted.

The error histogram is one of the measurements that evaluates the performance of the ANN on the colour fundus images of the DR data set to diagnose its early stages before blindness. In each epoch, the network records the difference between the actual and expected values during each phase based on instances. Each stage has its own colour, as shown in Fig 15. The blue colour indicates the performance of the ANN on the DR images during the training of the data set. Green colour when adjusting the weights and parameters of the ANN network on the DR images. The red colour shows ANN’s performance on the DR images while testing new samples. With the combined features of Dense-121 and the handcrafted features, ANN achieved the lowest error by the error histogram measure within 20 bins among values -0.9495 and 0.95. In contrast, with Alex combined features and handcrafted features, ANN achieved the lowest error by the error histogram measure within 20 bins among values -0.9494 and 0.9495.

#### 4.6.4. Gradient and validation checks

**Fig 16:**
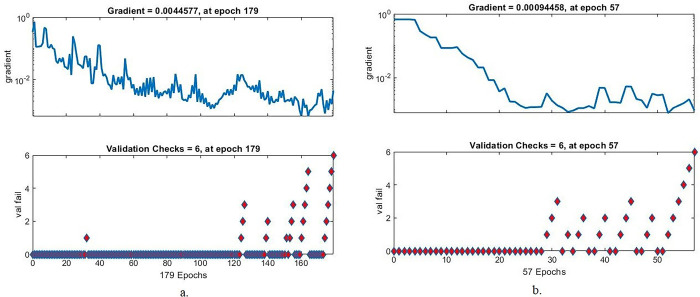
Display Gradient by ANN for early-stages DR dataset image diagnostics with radiomic features a. Dense-121 and handcrafted b. Alex and handcrafted.

Gradient and validation checks are one of the measurements that evaluate the performance of the ANN on the colour fundus images of the DR dataset to diagnose its early stages before blindness. In each epoch, the network runs gradient checks as well as validation checks and checks for failed values. With the combined features for Dense-121 and the handcrafted features, the ANN achieved the best evaluation in epoch 197 with a gradient value of 0.0044577 and achieved validation of 6. In contrast, with the combined features for Alex and handcrafted features, the ANN achieved the best evaluation in epoch 57 with a gradient value of 0.00094458 and achieved validation 6, as shown in Fig 16.

## 5. Discussion of the system’s performance

In this work, three integrated methodologies were developed; each methodology has two integrated systems with different techniques for image diagnosis of the DR dataset. All systems aim to achieve promising results for early prediction of the stages of development of DR before reaching the proliferative stage in which the patient becomes blind. Due to the similarity of the characteristics of DR images in the early stages, this study focused on analyzing DR images using many methods to extract features and combine them in the same vectors to generate vectors with important features from several methods. Due to DR images’ low contrast and noise, images have been improved with the same filters for all systems. The classes of the DR data set have been balanced, and overfitting was overcome by the data augmentation method for all systems.

The provided information describes different methodologies and their corresponding accuracies in diagnosing diabetic retinopathy (DR) and predicting its developmental stages using artificial neural networks (ANN) and various feature extraction techniques.

Here are some explanations for the differences in performance between the proposed methodologies: Dense-121 and Alex are different models with different architectures and capabilities. Dense-121 is a deeper model with more parameters than Alex, which has allowed it to learn more complex features from the DR images. Combining features from Dense-121 and Alex has improved the accuracy by providing the ANN with a more comprehensive set of features to work with. PCA has also helped to improve the accuracy by reducing the dimensionality of the feature space, which can make it easier for the ANN to learn the relationships between the features. Handcrafted features are created to capture specific features of DR images, which have helped the ANN to better understand the images and make more accurate predictions. The combination of deep learning features with handcrafted features has led to higher accuracy in the third methodology. The ANN achieved an accuracy of 98.6% with Dense-121 and handcrafted features and 99.1% with Alex and handcrafted features. The combination of the deep learning features with handcrafted features, specifically designed to capture certain characteristics of DR images, likely led to the higher accuracy in this methodology.

**Fig 17:**
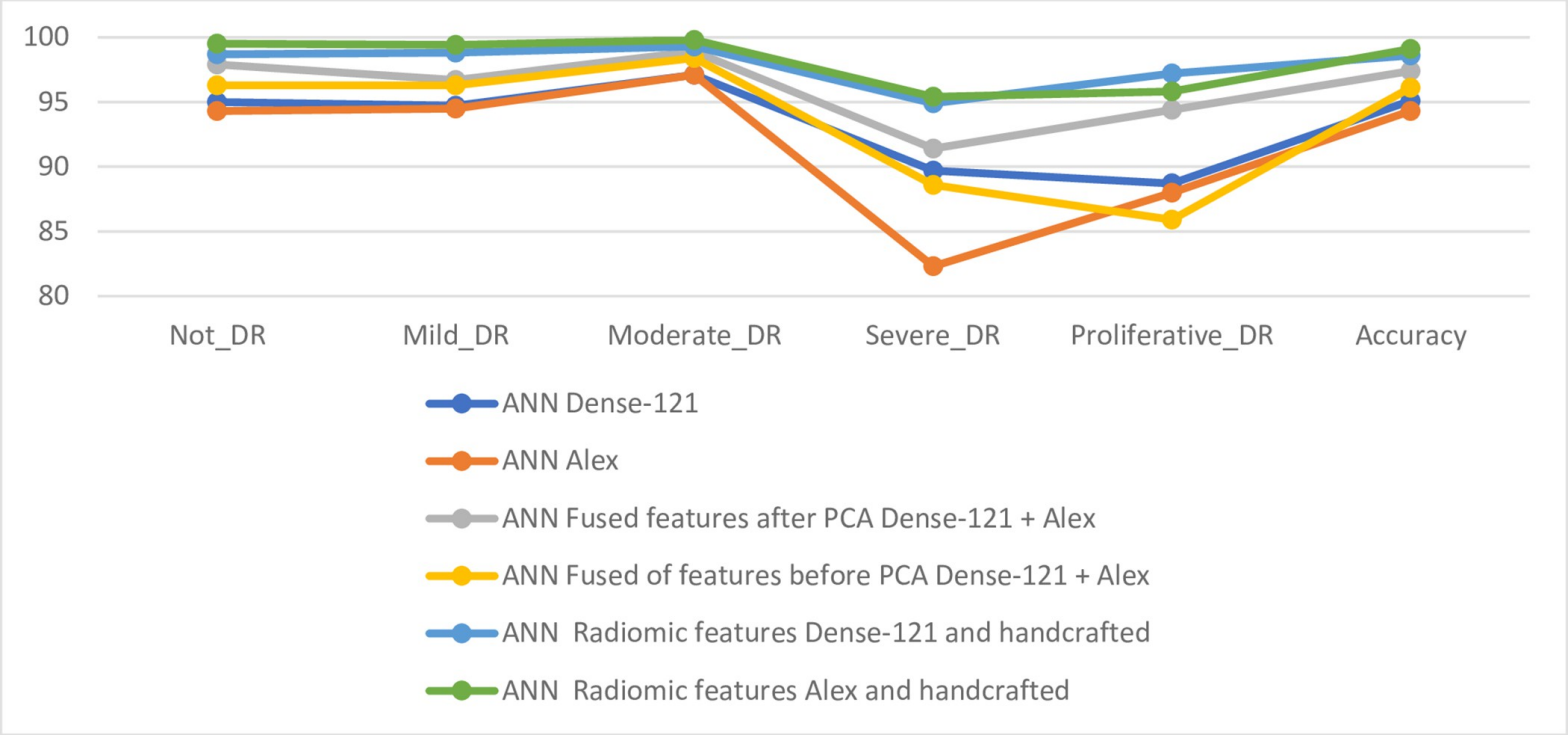
Displaying the performance of the ANN for diagnosis of images in the DR dataset for early prediction of its development stages.

Table 7 and Fig 17 summarize the results of DR image diagnostics for all systems to predict the early stages of development before blindness. The table shows the overall accuracy of all systems. It is noted that the best accuracy was achieved when feeding the hybrid features of the Alex model and the handcrafted features to the ANN network, which reached an accuracy of 99.1%. For stages (classes) Not_DR, Mild_DR, Moderate_DR, and Severe_DR, the ANN with the combined features of Alex and handcrafted features achieved the best diagnosis with accuracy of 99.5%, 99.4%, 99.8% and 95.4%, respectively. Whereas for the Proliferative_DR class, the ANN with the combined features of Dense-121 and handcrafted features achieved the best diagnosis with an accuracy of 97.2%.

**Table 7:** Results of DR dataset image diagnostic for early prediction of DR development stages for each class and the last column the overall accuracy.

Techniques		Features	Not_DR	Mild_DR	Moderate_DR	Severe_DR	Proliferative_DR	Accuracy
ANN		Dense-121	95	94.7	97.1	89.7	88.7	95.1
		Alex	94.3	94.5	97.1	82.3	88	94.3
ANN	Fused features after PCA	Dense-121 + Alex	97.9	96.7	98.9	91.4	94.4	97.4
	Fused of features before PCA	Dense-121 + Alex	96.3	96.3	98.4	88.6	85.9	96.1
	Radiomic features	Dense-121 and handcrafted	98.7	98.8	99.3	94.9	97.2	98.6
		Alex and handcrafted	99.5	99.4	99.8	95.4	95.8	99.1

Saxena et al. [7], Abhishek et al. [8], Borys et al. [9], Muhammad et al. [10], Shu et al. [12], Alexandr et al. [13], Fouzia et al. [14], Gadekallu et al. [15], Ludwig et al. [16], Ayushi et al. [17], Renukadevi et al. [19]: These studies focused on various deep learning and machine learning approaches, using different architectures and techniques for retinopathy diagnosis. Specific performance metrics vary across the studies, but accuracies range from 65.6% to 96.6%. Gadekallu et al. [21]: Utilized the GWO algorithm to select optimal features and achieved a sensitivity of 91% and an accuracy of 97.3% in diagnosing DR. This study combines radiomic features from Dense-121 and handcrafted features (DWT, LBP, FCH, and GLCM), as well as hybrid features from the Alex model and handcrafted features. The ANN model achieved high accuracies of 98.6% and 99.1% using the combined features, outperforming the other studies in terms of accuracy. Overall, this proposed systems demonstrates superior performance compared to the previous studies, achieving high accuracies in diagnosing DR by combining radiomic and handcrafted features.

## 6. Conclusions

Early detection of the stages of development of DR is necessary to avoid its progression to its final stages and blindness. This work presents the development of three novel approaches with six different techniques. All colour fundus images have been subjected to image enhancement and increasing contrast ROI through filters. All features extracted by CNN models (Dense-121 and Alex) were fed to the PCA method to select important features and reduce their dimensions. The first approach to the diagnosis of the images of the DR dataset by ANN with significant low dimensional features of Dense-121 and Alex models separately. The second approach to the diagnosis of the images of the DR dataset by ANN with combined features of Dense-121 and Alex models after dimension reduction and before dimension reduction by PCA. The third approach to the diagnosis of the images of the DR dataset by ANN with the radiomic features. The radiomic features are two matrices of radiomic features that are Dense-121 model merging with handcrafted features, and the Alex model merging with handcrafted features. All the systems reached promising results for the early detection of the DR. With the radiomic features of the Alex model and the handcrafted features. The ANN achieved a sensitivity of 97.92%, an AUC of 99.56%, an accuracy of 99.1%, a specificity of 99.4% and a precision of 99.06%.
